# 3.0T MRI for long-term observation of lung nodules post cryoablation: a pilot study

**DOI:** 10.1186/s40644-017-0131-7

**Published:** 2017-12-01

**Authors:** Jing Li, Jinrong Qu, Hongkai Zhang, Yingshu Wang, Lin Zheng, Xiang Geng, Yan Zhao, Hailiang Li

**Affiliations:** Department of Radiology, the Affiliated Cancer Hospital of Zhengzhou University, Henan Cancer Hospital, 127 Dongming Road, Jinshui District, Zhengzhou, Henan Province 450008 China

**Keywords:** Lung cancer, Magnetic resonance imaging, Percutaneous cryotherapy

## Abstract

**Background:**

The purpose of this study was to use serial magnetic resonance imaging (MRI) examinations to observe changes in malignant lung tumors over time post-cryoablation.

**Methods:**

The study protocol was approved by Institutional Review Board, and written informed consent was obtained from each participant in accordance with the Declaration of Helsinki. Patients with primary or metastatic lung tumors eligible for cryoablation were included in this prospective study. Cryoablation was performed according to standard procedures. Unenhanced and dynamic contrast-enhanced MRI scans were performed pre-cryoablation and at 1 day, 1 week, and 3-, 6-, and 12 months after cryoablation. At each time point, the signal intensity of the ablated zone on both T_1_WI and T_2_WI images, and volume and characteristics of the ablation zone were examined, and changes over time analyzed.

**Results:**

A total of 26 nodules in 23 patients were included in the study. The mean patient age was 53.7 ± 13.6 years, and 57.7% were males. Ablation zone volume increased to 1 week after the procedure, and then returned to baseline by 3 months. Cavitation post-cryoablation was found in 34.6% (9/26) of the nodules 1 month after treatment. Two types of time-signal intensity curves post-cryoablation were found: a straight line representing no definite enhancement from 1-day to 1-month, and an inflow curve representing mild delayed enhancement from month 3 to month 12. Local progression was associated with an incomplete hypointense rim around the ablation zone and absence of cavitation post-treatment.

**Conclusions:**

Characteristic changes are present on MRI after cryoablation of lung tumors. A complete hypointense rim and cavitation may be signs of adequate treatment and that local tumor progression is less likely.

**Electronic supplementary material:**

The online version of this article (doi:10.1186/s40644-017-0131-7) contains supplementary material, which is available to authorized users.

## Background

Lung cancer is the most common cancer worldwide, and is the leading cause of cancer-related death in both men and women [1]. Surgical resection is the standard treatment for early-stage patients. However, patients with advanced stage disease and those with significant comorbidities may not be eligible for surgery. Metastases is the second most common pulmonary malignancy, and resectability depends on many factors [2]. Nonsurgical candidates are usually managed with systemic chemotherapy; however, side effects can be severe and not all tumors respond to chemotherapy [3–5].

Cryoablation of a tumor can reduce its size, or completely eliminate it, and can thus alleviate symptoms and improve survival [1, 6]. Cryosurgery is commonly used for the treatment of prostate and liver tumors [7, 8]. Although percutaneous cryoablation of tumors in the thorax was first reported in 2005 [9], and it has been shown to be safe and effective for lung tumors [3, 5, 10], it is still not widely used for lung malignancies [11]. No tissue is removed during cryoablation; thus, imaging is necessary to determine if the extent of ablation is adequate, and to determine how the lesion has responded to the treatment. Imaging studies are mainly utilized for intra-operative guidance, and there is only 1 report on application of computed tomography (CT) for determination of the ablated zone and the examination of serial post-ablative changes [12]. While the study showed that CT was useful for examining changes following cryoablation, the large radiation dose from multiple CT scans cannot be ignored.

Magnetic resonance imaging (MRI) has advanced such that artifacts due to breathing and heart function can be reduced or eliminated, and MRI is frequently used for the diagnosis of thoracic disease [13, 14]. MRI has been shown to clearly reflect changes in the ablation zone after radiofrequency ablation of liver lesions [15]. Unlike CT, MRI is not associated with ionizing radiation. However, there is currently only 1 report of MR-guided cryoablation for lung cancer [16], and data of long-term changes of the ablation zone using MRI are not available.

We hypothesized that MRI can identify changes in the ablation zone after radiofrequency ablation of lung tumors, and thus may be useful for monitoring treatment effectiveness. Thus, the purpose of this study was to use serial MRI examinations to observe changes in malignant lung tumors over time post-cryoablation, and correlate these changes with the effects of treatment.

## Methods

### Patients

The study protocol was approved by Institutional Review Board, and written informed consent was obtained from each participant in accordance with the Declaration of Helsinki.

This prospective study included patients with primary or metastatic lung cancer who received cryoablation from December 2013 to December 2015. Malignancy was diagnosed by a tissue specimen obtained via percutaneous or transbronchial lung biopsy, or clinical changes in the nodule such as rapid enlargement or glucose uptake on positron emission tomography (PET/CT). Other inclusion criteria were 1) newly diagnosed and had received no prior treatment; 2) were judged able to tolerate general anesthesia and the cryoablation procedure; 3) had a single nodule in the target lobe; 4) nodule size <4 cm and no invasion into the adjacent structure; and 5) can complete the entire MRI scanning plan. Patients were excluded if 1) they had more than 1 tumor in the target lobe or had received prior therapy; 2) image quality was too poor to meet diagnostic requirements; 3) significant comorbidities or vital organ failure were present; and 4) nodule size >4 cm, or invasion into mediastinal structures, chest wall, segmental bronchi, vessels, or nerves.

During the post ablation follow-up period, progression was diagnosed by biopsy only if the patient was able to tolerate the procedure, or changes in the nodule such as rapid enlargement or increased glucose uptake were identified on positron emission tomography (PET/CT).

### Cryoablation protocol

Cryoablation was performed under general anesthesia and CT guidance. All procedures were performed with a percutaneous cryoablation device utilizing 1.7 mm diameter cryoprobe and an Ar-He cryosurgery unit (CryoHit type; Galil Medical, Yokneam, Israel). The target lung nodule was identified, and a 3-dimensional (3D) treatment plan was designed based on 16 slice helical CT images (Light Speed; GE Medical Systems, Milwaukee, WI). The criteria for effective treatment were an ice hockey puck zone 1 cm or more from the edge of the target nodule, and the frozen range after 1 single procedure covered more than 80% of the lesion. The needle interval was set to 2 cm when using 2 needles so that the ice ball produced by each needle slightly overlapped and formed a seamless large frozen ball. The number of needles used depended on the size of the tumor.

After the skin was prepped and draped, 1% lidocaine was injected subcutaneous from the skin to the pleura. A 21-gauge guide needle was inserted along the planned optimized path using scout CT images. The needle position was confirmed in the targeting area by repeat CT scanning, then the 17-gauge stainless steel cryoablation probes consisting of an external sheath and inner-guide needle was inserted. The guide needle was removed after confirming the position, and the cryoprobe was introduced through the external sheath. The needle always penetrated the tumor, and the tip of the needle was placed at the far end of the lesion. Freeze-thaw cycles were then performed. A single cycle consisted of a 10 min freezing period in which the local temperature decreased to minus 170 °C due to argon gas rapid expansion, and a 2-min re-warming period in which the local temperature increased to plus 20 °C as a result of helium rapid expansion. Generally, a single procedure consisted of 2 freeze-thaw cycles; however, in the case of large tumors multiple cycles were performed.

### MRI scanning program and parameters

All patients received chest unenhanced and dynamic enhanced MR scans pre-cryoablation, and at 1 day, 1 week, and 1, 3, 6, and 12 months after the procedure. Scanning was done with a 3.0T MR scanner (SignaHDx; GE Healthcare, Waukesha, WI), using a TORSO coil. Patients were in the supine position, foot ahead. Imaging was performed from the apex to the top of the diaphragm, including the target lung nodule. Six different sequences were used, and the sequences and parameters are summarized in Additional file 1: Table S1.

### Image analysis

The size of the ablation zone was measured in 3 dimensions on each imaging study. In prior studies, the post-ablation zone was characterized by regional ground-glass opacity on CT images [2, 12, 17]. As MRI characteristics of the ablation zone for pulmonary nodules are not available, we referred to studies of MRI characteristics after cryoablation for liver tumors [18, 19]. The ablation zone was defined as an area of high signal intensity on both T1WI and T2WI images, and no enhancement, which histologically represents the region of coagulative necrosis. Tumor volume (lesion length × lesion width × lesion height)/2) was recorded for each ablation zone. Each diameter was measured 3 times by an independent radiologist, and the average value was used for further calculations.

All MRI images at each time point were analyzed by 2 radiologists with more than 5 years of MRI diagnostic experience. Analyses were done independently and blindly. If there were inconsistencies at the final review of all of the analysis, a third highly qualified radiologist with more than 10 years of MRI diagnostic experience reviewed the imaging studies and made the final determination.

**Fig. 1:**
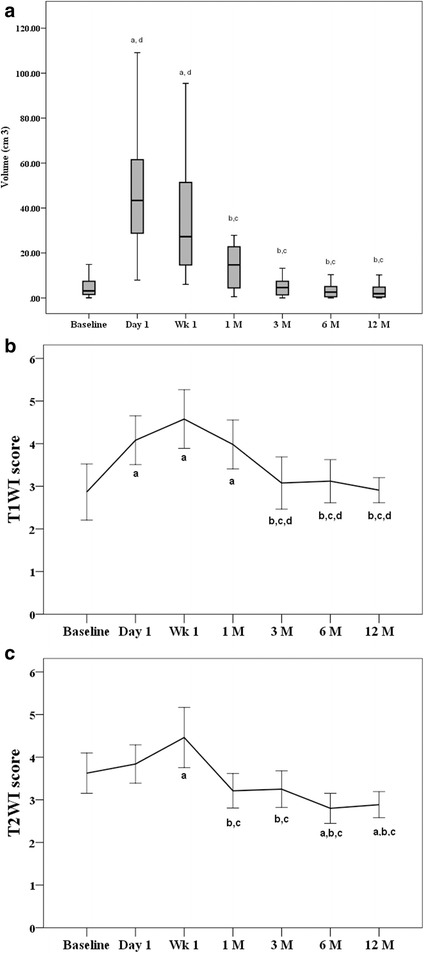
Changes in volume (**a**) and T_1_WI (**b**) and T_2_WI score (**c**) during the study period (*n* = 26 nodules). Nodule volume data are presented as a box plot: 1st quartile (top of the box), median (line shown in box), 3rd quartile (bottom of the box), and examined Friedman’s test. T_1_WI and T_2_WI data are presented as mean ± standard deviation, and tested by linear mixed model. Letters denote significant difference between the given time and baseline^a^, day 1^b^, week 1^c^, or 1 month ^d^ (*p* < 0.002). Volume (cm^3^)

Signal intensity of the ablation zone on T_1_WI and T_2_WI images were observed carefully, and changing characteristics and time-signal intensity changes were recorded. There are no standard criteria or quantitative parameters of T_1_WI and T_2_WI sequences for evaluating lung nodule post-cryoablation. However, a 5-point grading scale method has been proven to be effective and reliable for subjectively evaluating image quality, and to compare different technologies in tumor diagnosis [20, 21]. Thus, we applied a 5-point grading scale ranging from 1 (low) to 5 (high) on both T_1_WI and T_2_WI images for scoring signal intensity of the ablation zone. The scoring criteria were: On T_1_WI images, lung parenchyma background was scored as 1 point, muscle signals as 3 points, fat tissue signal intensity as 5 points, signal intensity between lung parenchyma and muscle as 2 points, between muscle and fat tissues as 4 points (Fig. 1a). On T_2_WI images, lung parenchyma background was scored as 1 point, muscle signals as 3 points, water-like high signal intensity as 5 points, signals between parenchyma and muscles as 2 points, and between muscle and water as 4 points (Fig. 1b). The ablation zone on T_1_WI and T_2_WI images at 1-day post-cryotherapy often showed heterogeneous signal intensity. When this occurred, the scores were determined by the dominant signals at the largest part of the ablation zone. Accompanying signs were also observed, including inflammation, atelectasis, and pleural effusion. Inflammation was identified as a large flake appearance with blurred margins, slightly high T_1_WI and T_2_WI signals with bronchial low signal intensity inside, and marked enhancement and blood vessels penetrating signs. Atelectasis was identified as a reduced wedge shape of a segment or lobe, slightly high signal intensity on T_1_WI and T_2_WI images without a bronchial low signal intensity inside, and significantly enhanced with a clear and straight border. Pleural effusion was identified by a crescent or ribbon-like intra-thoracic water-like signal intensity.

Dynamic enhanced sequences, including mask phase, were transmitted to a Work Station 4.5, and processed by the SER software package. A region of interest (ROI) was placed in the aorta, and in the largest cross-section of the ablation zone on the same slice. Time-signal intensity curves were automatically generated.

## Statistical analysis

Mean and standard deviation were computed for age, nodule volume, T_1_WI, and T_2_WI, and age range was reported as well. Categorical variables were summarized as frequency and percentage. Inter-rater reliability for T_1_WI and T_2_WI analysis was examined by weighted Kappa. Five levels of agreement were defined according to the Kappa statistic: poor (κ < 0.2), fair (0.2 ≤ κ < 0.4), moderate (0.4 ≤ κ <0.6), good (0.6 ≤ κ < 0.8), and very good (0.8 ≤ κ ≤ 1.00). Nodule volume at various time points were presented as a box plot: 1st quartile (top of the box), median (line shown in box), 3rd quartile (bottom of the box). Data on nodule volume were examined by Friedman’s test, while the time trends of T_1_WI and T_2_WI signal intensity were presented as line charts and examined using a linear mixed model. If the time effect was significant, post-hoc tests were implemented using Bonferroni’s correction method. To compare differences in T_1_WI and T_2_WI signal intensity between patients with and without local tumor progression, the Mann-Whitney U test was also performed. A value of *p* < 0.05 was considered statistical significant; the significance level was adjusted to 0.002 (0.05/21) if post-hoc tests were required. All statistical analyses were performed with PASW statistical software (version 21.0, IBM Corp., Armonk, NY, USA).

## Results

### Patients

Data of 26 nodules from 23 patients were used for the analysis (Table 1). The mean patient age was 53.7 ± 13.6 (range: 25–78) years, and 57.7% were males. Of the 23 patients, 12 were lost to follow-up at 18 months, and another 5 were lost at 24 months.

**Table 1:** Characteristics of 26 nodules in 23 patients

Age, years		53.7 ± 13.6 (25–78)
Gender	Female	11 (42.3)
	Male	15 (57.7)
Metastasis	No	11 (42.3)
	Yes	15 (57.7)
Histological type	Adenocarcinoma	22 (84.6)
	Squamous cell carcinoma	4 (15.4)
Local progression	No	20 (76.9)
	Yes	6 (23.1)

Age reported as mean ± standard deviation with (range); other data reported as number (percentage)

### Volume change characteristics

Changes in nodule volume through the study period are illustrated in Fig. 1a. The volume was increased at post-procedure day 1, then became smaller gradually at 1 week and 1 month, and then reduced in size to the baseline value at 3 months and remained at that size until the 12th month examination.

### Cavity formation

Other MRI findings such as cavitation and complications are summarized in Table 2. Cavitation post-cryoablation was found in 34.6% (9/26) of the nodules 1 month after treatment, but no further cavitation was noted as time progressed. Six of the 9 cases of cavitation were resolved at 3 months, and the others were resolved by 6 months. Interestingly, none of the patients with cavitation post-cryoablation developed local tumor progression.

**Table 2:** MRI findings and complications (26 nodules)

	Post-cryoablation cavitation	Time-intensity curve type^a^	Consolidation	Atelectasis	Pneumothorax
Baseline	0	Vary	0	0	0
Day 1	0	1	42.3% (11/26)	30.8% (8/26)	26.9% (7/26)
Week 1	0	1	26.9% (7/26)	7.7% (2/26)	3.8% (1/26)
Month 1	34.6% (9/26)	1	7.7% (2/26)	3.8% (1/26)	0
Month 3	11.5% (3/26)	2	0	0	0
Month 6	0	2	0	0	0
Month 12	0	2	0	0	0

^a^Two classifications of time-intensity curve: 1, no definite enhancement as a straight line; 2, mild delayed enhancement as an inflow curve

Consolidation, atelectasis, and pneumothorax were found in 42.3%, 30.8%, and 26.9% of nodules 1 day after the treatment, respectively. No consolidation or atelectasis was observed after 3 months, and pneumothoraces were resolved by 1 month.

### Changes of signal intensity

The inter-rater reliability with regard to observation of T_1_WI and T_2_WI signal intensity are reported in Table 3. Regardless of time, the inter-rater reliability of T_1_WI and T_2_WI were good or very good, except that T_2_WI measured at month 6 only achieved moderate reliability. Changes in T_1_WI and T_2_WI are illustrated in Fig. 1b and c. The T_1_WI score increased from 2.87 at baseline to the peak of 4.58 at week 1, then declined to around 3 after 3 months (Fig. 1b). A similar trend was found for T_2_WI, except the T_2_WI score significantly decreased to 3.2 at 1 month, and then leveled off to around 2.9 at 6 months (Fig. 1c).

**Table 3:** Inter-rater reliability for T_1_WI and T_2_WI

	T_1_WI		T_2_WI	
Time	Weighted Kappa	95% CI	Weighted Kappa	95% CI
Baseline	0.943	(0.831–1.000)	0.824	(0.593–1.000)
Day 1	0.750	(0.446–1.000)	0.800	(0.533–1.000)
Week 1	0.878	(0.706–1.000)	1.000	(1.000–1.000)
Month 1	0.930	(0.794–1.000)	0.885	(0.665–1.000)
Month 3	0.875	(0.704–1.000)	0.898	(0.702–1.000)
Month 6	0.829	(0.595–1.000)	0.500	(0.076–0.924)
Month 12	1.000	(1.000–1.000)	0.776	(0.357–1.000)

*CI* confidence interval

### Enhancement characteristics

**Fig. 2:**
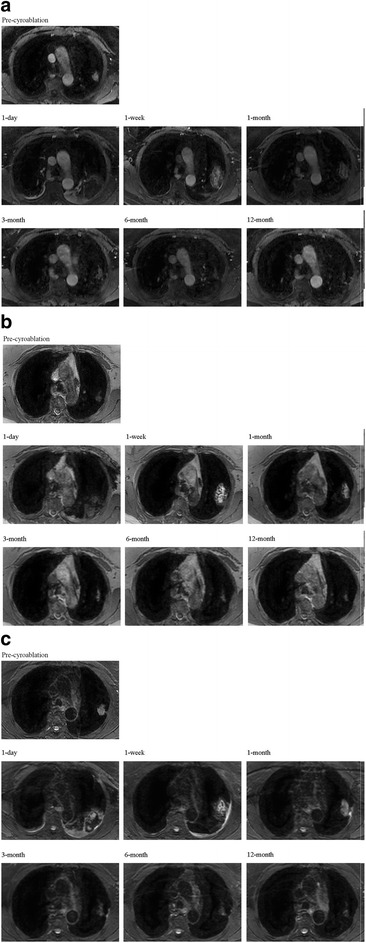
Post-contrast images of venous phase (**a**), T_1_WI (**b**), and T_2_WI (**c**) of a non-progressive lesion. At the early stage (1 day, 1-month and 3-month), there was no definite enhancement in the ablation zone; later, mild enhancement was noted. Similarly, at the early stage the time-signal curve was a straight line type, and later became an inflow type

**Fig. 3:**
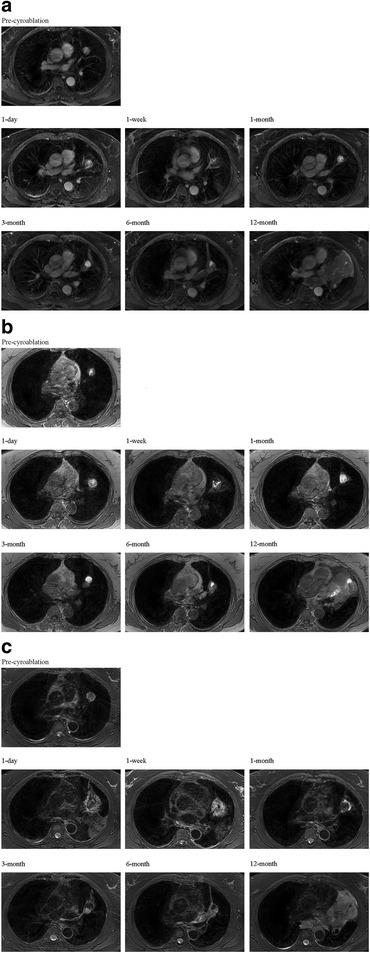
Post-contrast images of venous phase (**a**), T_1_WI (**b**), and T_2_WI (**c**) of a progressive lesion. Progression was noted at 3 months postoperatively. Images obtained at 1 week and 1 month postoperatively showed an unclear and incomplete hypointense rim, especially the T_2_WI images

Lung nodules exhibited different enhancement characteristics pre-cryoablation. The majority (73.1%, 19/26) exhibited moderate persistent enhancement, and the time-intensity curve was plateau (type II). However, there were 2 types of time-signal intensity curves post-cryoablation: a straight line representing no definite enhancement from 1-day to 1-month, and an inflow curve representing mild delayed enhancement from month 3 to month 12 (Fig. 2). Images of a progressive lesion are shown in Fig. 3.

### Local tumor progression

At 12 months after treatment, 4 nodules showed local tumor progression. At 18 months after treatment, and additional 2 nodules developed local progression. A difference in T_1_WI signal intensity between patients with and without local tumor progression was found; however, the difference was no longer statistically significant after 1 week. A difference in T_2_WI signal intensity was also noted after treatment, but the difference became none significant at 6 months. At 3 months after treatment the ablation zone in 22.7% (5/22) of nodules remain round or nodule-like in the non-progression group, and at 12 months 77.3% (17/22) of ablation zones had streaks and or a patch shape.

Of 6 patients that developed local tumor progression, 4 were male and adenocarcinoma was the histological type in 4 cases. Complications like consolidation, atelectasis, and pneumothorax occurred within 1 week to 3 months after treatment (Table 4). Two patients received treatment with embedded ^125^I particles, 2 a second cyroablation, and 2 local radiotherapy. The ablation zone in the 6 patients with progression showed an incomplete ring at 1 week and 3 months on T_1_WI and T_2_WI images, suggesting that this finding early post-cryoablation may be an indicator for progression.

**Table 4:** Characteristics of 6 patients with local tumor progression

	Patient 1	Patient 2	Patient 3	Patient 4	Patient 5	Patient 6
Age, years	25	57	62	56	55	57
Gender	M	F	M	M	F	M
Number of treated nodules	1	1	1	1	1	1
Histological type	AD	SCC	AD	AD	AD	SCC
Metastasis	Y	N	Y	Y	N	N
Volume, cm^3^						
Baseline	1.46	14.91	5.66	1.52	0.25	4.20
Day 1	29.71	51.89	124.71	29.74	46.37	35.34
Week 1	16.09	47.05	95.47	22.46	26.04	26.84
Month 1	7.15	23.25	27.84	8.54	20.39	9.98
Month 3	1.37	7.95	8.72	1.37	5.92	7.30
Month 6	0.52	5.83	N/A	1.82	4.54	3.56
Month 12	0.46	4.83	N/A	N/A	N/A	N/A
T1WI						
Baseline	2	2	3	3	2	2
Day 1	4	5	4	4	5	5
Week 1	5	5	5	5	5	5
Month 1	4	5	4	4	4	5
Month 3	4	3	3	3	3	3
Month 6	4	3	N/A	3	3	3
Month 12	3	3	N/A	N/A	N/A	N/A
T2WI						
Baseline	4	4	4	4	3	4
Day 1	4	4	4	4	4	4
Week 1	5	5	5	5	5	5
Month 1	3	3	3	3	3	3
Month 3	3	4	4	4	4	4
Month 6	3	3	N/A	3	3	3
Month 12	3	3	N/A	N/A	N/A	N/A
Post-cryoablation cavitation	N	N	N	N	N	N
Consolidation						
Baseline	N	N	N	N	N	N
Day 1	Y	Y	Y	Y	Y	Y
Week 1	Y	Y	Y	Y	Y	Y
Month 1	N	Y	Y	Y	Y	Y
Month 3	N	N	Y	Y	Y	N
Month 6	N	N	N/A	N	N	N
Month 12	N	N	N/A	N/A	N/A	N/A
Atelectasis						
Baseline	N	N	N	N	N	N
Day 1	Y	Y	Y	Y	Y	Y
Week 1	Y	Y	Y	Y	Y	Y
Month 1	N	Y	Y	Y	Y	Y
Month 3	N	N	Y	N	N	N
Month 6	N	N	N/A	N	N	N
Month 12	N	N	N/A	N/A	N/A	N/A
Pneumothorax						
Baseline	N	N	N	N	N	N
Day 1	N	Y	Y	Y	Y	N
Week 1	N	N	Y	N	Y	N
Month 1	N	N	N	N	N	N
Month 3	N	N	N	N	N	N
Month 6	N	N	N/A	N	N	N
Month 12	N	N	N/A	N/A	N/A	N/A

*AD* adenocarcinoma, *N* no, *N/A* not available, *SC* squamous cell carcinoma, *Y* yes

## Discussion

A number of studies have shown that cryoablation is an effective treatment for primary lung tumors and pulmonary metastasis [3, 4, 22]. However, familiarity with the post-procedural findings, and the ability to discriminate them from local progression, is critically important.

Our study showed there are characteristic changes of lung nodules after cryotherapy. There is an early increase in volume of the ablated zone that peaks at 1 day after the procedure, remains consistent to 1 week, and then gradually declines to the baseline size at 3 months. The volume increase at 1 day is secondary to ice ball formation and acute lung changes in the target region. However, the reason why the signal is heterogeneous at 1 day post-cryoablation is not clear. Heterogeneous signals can reflect post-cryoablation complications; a high signal on T_1_WI and T_2_WI images can be due to alveolar hemorrhage, which is suggestive of small vessel rupture, whereas a low signal in the outer periphery may be the result of alveolar wall collapse and small bronchi rupture. In an animal study, Romaneehsen et al. [23] showed that a volume decrease at 1 week after cryoablation was associated with a reduction or disappearance of a low signal region in the outer periphery, which is suggestive that tissue repair begins in the outer region. A signal increase in the core of the ablated zone has been shown by animal experiments to be secondary to coagulation necrosis [23, 24]. This findings has also been found in radiofrequency ablation in the liver [15].

The persistent initial volume increase is secondary to the mobilization of macrophages and neutrophils into the cryoablation zone as part of the post-cryoablation immunologic cascade, which leads to tumor lysis and ensuing tissue repair [24]. Subsequently, as the concentration of cellular debris, macrophages, and neutrophils decreases, there is steady reduction in the volume of the cryoablation zone [24], as noted in our study. It is unclear why there is an increase of T_2_WI signal intensity in the ablated zone at 1-month post-cryoablation, as there is no relevant literature on the topic. It may secondary to stromal hyperplasia, which is abundant in newly developing small vessels and new fibrous tissue, thus reflecting tissue repair [6, 24]. The continuous decrease of signal intensity on T_1_WI and T_2_WI images in the ablated zone from 3 months onward may represent repair and fibrosis.

In our study, at 1 day, 1- week, and 1 month post-cryoablation, the ablated zone showed no enhancement, which may be due to complex changes related to cell necrosis [15, 23]. From 3 months onward mild enhancement was note in nodules that did not develop local progression. This is consistent with CT findings of lung nodules post-ablation [6, 12].

The local progression rate in our study during a 12-month follow-up period was lower than that reported by Liu et al. [16] and Inoue et al. [10] Most nodules (73.7%) in our study had a diameter < 30 mm, and approximately 30% had a diameter of 30–40 mm; thus, they were relatively small. The low progression rate in this study may due to the limited sample size, size of the nodules, or the histological cancer types.

The presence of hypointense rim as a “ring” appears to be critically important, as suggested from study of radiofrequency ablation [25]. In this study, the hypointense rim was present at 1-week and 1-month post-cryoablation, and was a complete rim in 84.6% of nodules and incomplete in 15.4%. Although statistical comparative analysis in the progression group could not be performed due to the small sample size, all cases with local progression exhibited an incomplete ring at the ablation zone at 1 week and 3 months on T_1_WI and T_2_WI images, suggesting that this finding early post-cryoablation may be an indicator for progression.

Studies of radiofrequency ablation indicate the presence of cavitation is a very important predictor of prognosis [6, 12]. We found cavitation in approximately 30% of nodules after treatment, which is similar to the 35% reported by Ito et al. [26], but lower than the 53% reported by Chaudhry et al. [12]. The spectrum of pulmonary nodules in our study was similar to that of the study by Ito et al., but different from that of Chaudhry et al. who only included patients with stage I non-small cell lung cancer. None of the patients who developed tumor progression had evidence of cavitation, suggesting that cavitation may predict a good clinical outcome. In our study, the ablated zone in approximately 77% of patients without local progression eventually developed streaks and a patch shape, and the other cases remained round or nodule shaped. Chaudhry et al. [12] found that shape change in the ablated occurred up to 6 months after treatment, and then remains stable to 12 months after cryoablation.

While the current study focused on MRI changes, other studies examined CT and PET/CT for the evaluation of nodules post ablation. Abtin et al. [6] examined CT, PET, and dual-modality imaging with combined PET and CT (PET/CT) for their value in identifying partial ablation, tumor recurrence, and progression in lung nodules treated with RFA. The authors divided post ablation into 3 periods: early (up to 1 week), intermediate (> 1 week to 2 months), and late (> 2 months). Imaging features that were suggestive of residual or recurrent disease included 1) increasing contrast agent uptake in the ablation zone (>180 s on dynamic images), nodular enhancement >10 mm, central enhancement >15 HU, and enhancement greater than baseline any time after ablation; 2) growth of the ablation zone after 3 months as compared with baseline, peripheral nodular growth, and change from ground-glass opacity to solid opacity; 3) increased metabolic activity beyond 2 months, residual activity centrally or at the ablated tumor, and development of nodular activity.

There are limitations to what we consider a preliminary study. The sample size was small, the nodules were extremely heterogeneous, i.e., they were both primary and metastatic cancer and the histological type varied, and because of patients lost to follow-up only data up to 12 months could be analyzed. We could not correlate MRI findings with histopathological changes as performing repeat biopsies solely for the purposes of research is not ethical. We did not examine DWI sequence data and ADC values, which have been proven a reliable for the diagnosis of pulmonary nodules [2, 27], and evaluation of therapeutic response [21, 28]. These examinations were not performed because there were artifacts on DWI images at 1 day and 1 week post-cryoablation, and putting the ROI in the correct place was difficult due to cavity formation. The method of evaluating the effect of cryoablation, tissue changes, and local residual tumor and local tumor progression was subjective, and was based on CT observations of lung tumors and MRI observations of liver tumors, changes to lung tumors that have been cryoablated identified by MRI may not correspond. Positron emission thermography (PET)-CT may have provided greater value and more objective data with respect to examining serial changes in the tumors. However, PET is not generally covered by insurance (only 7 patients in the current study received PET-CT as part of their initial work-up), and our budget did not allow for us to perform the procedure on all patients, and there is the concern of ionizing radiation with repeated CT scanning. We did not compare MRI and CT data, as study has evaluated the value of CT, MRI, and histological examination after cryoablation of renal tumors and concluded that MRI is superior to CT [29].

## Conclusions

The results of this study showed there are characteristic changes that can be identified on MRI after cryoablation of lung tumors. A complete hypointense rim at 1 week and 1 month post-cryoablation, and cavitation in the ablation zone at 1 month post-cryoablation may be signs of adequate treatment and that local tumor progression is less likely. Though we consider this a preliminary study, the results suggest that MRI may be a good method to follow the results of cryoablation of lung tumors.

## Additional file

Additional file 1:
**Table S1**. Magnetic resonance sequences and parameters. (DOCX 18 kb)
