# Supplementary material for: 3.0T MRI for long-term observation of lung nodules post cryoablation: a pilot study
Source: Cancer Imaging. 2017 Dec 1;17:29. doi: 10.1186/s40644-017-0131-7 (PMC5709825; doi:10.1186/s40644-017-0131-7)
Supplement: Additional file 1: — Table S1. Magnetic resonance sequences and parameters. (DOCX 18 kb) [file 40644_2017_131_MOESM1_ESM.docx]

**Table S1. Magnetic resonance sequences and parameters**

| **Sequence** | **TR**  **（**ms**）** | **TE（**ms**）** | **Thickness**  **（**mm**）** | **Gap between sections**  **（**mm**）** | **NEX** | **FOV（**cm^2^**）** | **Matrix**  **（**[freq] ×[phase]**）** | **Flip angle** |
| --- | --- | --- | --- | --- | --- | --- | --- | --- |
| Breath-hold 3D spoiled gradient echo (BH 3D-SPGR) axial T_1_WI | 195 | 3 s | 4 | 0.4 | 0.75 | 40 × 40 | 280 × 192 | NA |
| Fast spin echo (FSE) of WATER Oax T_2_ ideal fat suppression T_2_WI | 8600 | 86 | 4 | 0.4 | 2 | 40 × 40 | 288 × 224 | NA |
| Respiratory gating RT coronal T_2_WI | 7800 | 85 | 6 | 1 | 1 | 38 × 38 | 288 × 224 | NA |
| Breath-hold SSFSE sagittal T_1_WI | 1871 | 120 | 6 | 1 | - | 35 × 35 | 384× 256 | NA |
| Axial dynamic contrast-enhanced (DCE) sequence with breath hold liver acquisition with volume acceleration (BH-LAVA) T_1_WI | 2.6 | 1.2 | 4 | 2 | - | 40 × 40 | 170 × 272 | 12° |

Abbreviation: NA=not available.

PS: A total dose of 0.1 mmol/kg body weight Gd-DTPA (Magnevist, Bayer Schering Pharma, Berlin, Germany) was injected via an antecubital vein via the pump injector (Medrad, Warrendale, Pennsylvania, USA) at the flow rate of 2.5 mL/s, after which 20 mL saline was injected at the same flow rate. There were 7 total phases including a mask phase obtained before contrast agent administration, and 6 enhanced images composed of 2 continuous scans (no intermediate time interval) at 12 s and 50 s, and 1 scan at 90 s and 1 scan at 150 s after contrast agent administration. A single complete enhanced scanning phase took 7-8 s, and provided 44 or 48 images, depending on the patient’s body size.
